# Impact of Atrial Lead Position on Functional Cardiac Parameters in Patients Requiring Dual-Chamber Pacemaker Implantation

**DOI:** 10.3390/jcm14072278

**Published:** 2025-03-27

**Authors:** Sarah X. Gharibeh, Valerie Jochmann, Istvan Szendey, Peter Jirak, Albert Topf, Dorothee Ladage, Uta C. Hoppe, Lars Eckardt, Emmanuel Chorianopoulos, Lukas J. Motloch, Robert Larbig

**Affiliations:** 1 Department of Internal Medicine II, Paracelsus Medical University, 5020 Salzburg, Austria; 2 Division of Cardiology, Hospital Maria Hilf, 41063 Moenchengladbach, Germany; 3 Department of Cardiology, RWTH Aachen University, 52074 Aachen, Germany; 4 Department of Internal Medicine, Danube Private University, 3500 Krems, Austria; 5 Division of Electrophysiology, Department of Cardiovascular Medicine, University of Münster, 48149 Münster, Germany; 6 Department of Internal Medicine II, Salzkammergut Klinikum, OÖG, 4840 Vöcklabruck, Austria; 7 Department of Cardiology, Kepler University Hospital, Medical Faculty, Johannes Kepler University, 4020 Linz, Austria

**Keywords:** DDD, pacemaker, Bachmann bundle pacing, right atrial appendage pacing, atrial fibrillation, p-wave duration, cardiology

## Abstract

**Background:** In patients requiring dual-chamber pacemaker (DDD) implantation, optimal atrial lead position remains a matter of debate. While most centers prefer implantation in the right atrial appendage position (Non-BB-P), due to a speculated favorable impact on atrial conduction characteristics, often, a Bachman bundle pacing (BB-P) is recommended. However, data investigating clinical outcomes in these patients are still rare. **Methods:** To evaluate this issue, in this retrospective single-center study, one-year clinical follow-up, pacemaker interrogations and available echocardiography findings in 301 consecutive patients (BB-P: age 76 ± 10 years, 46.7% female, n = 169; Non-BB-P: 77.6 ± 9 years, 50% female, n = 132, *p* = n.s.) scheduled for dual-chamber implantation were analyzed. **Results:** During follow-up, the incidence of atrial fibrillation (AF) remained similar in both groups (BB-P: 38.3%, n = 154 vs. Non-BB-P: 34.2%, n = 117 *p* = n.s.). However, we detected significantly more mode switch episodes in the BB-P group (BB-P: 51.9%, n = 154 vs. Non-BB-P: 38.8%, n = 116, *p* = 0.032). Furthermore, left ventricular functional parameters, including left ventricular ejection fraction (BB-P: 57.1 ± 8.4%, n = 60 vs. Non-BB-P: 56.0 ± 9.6, n = 45 *p* = n.s.) and incidence of diastolic dysfunction (BB-P: 55.2%, n = 67 vs. Non-BB-P: 38.3%, n = 47, *p* = n.s.), as well as the rate of left (BB-P: 58.8%, n = 68 vs. Non-BB-P: 42.0%, n = 50, *p* = n.s.) and right atrial dilatation (BB-P: 27.9%, n = 68 vs. Non-BB-P: 28.0%, n = 50 *p* = n.s.), were not significantly affected by the atrial lead position. However, stimulated p-waves were significantly shorter in BB-P vs. Non-BB-P (BB-P: 132.9 ± 23.7 ms, n = 127 vs. Non-BB-P: 139.6 ± 23.4 ms, n = 93, *p* = 0.031). **Conclusions:** In patients requiring dual-chamber implantation, the position of the atrial lead significantly altered atrial conduction, but this did not seem to affect left ventricular function parameters or the occurrence of atrial fibrillation within our follow-up period. Interestingly, we even detected more mode switch episodes in the BB-P group, hinting at an even proarrhythmic potential of BB-P. On the other hand, we found a decreased ventricular stimulation percentage in BB-P vs. Non-BB-P. Further studies should investigate the impact of Bachmann bundle pacing on clinical outcomes.

## 1. Introduction

The optimal lead placement of the atrial electrode in patients that require DDD pacing has been discussed controversially. Bachman bundle pacing (BB-P) has been suggested to possess both hemodynamically and electrophysiological preferable properties in comparison to low-atrial septum pacing or pacing of the right atrial appendage (Non-BB-P) [1,2,3]. Specifically, the Bachmann bundle, located at the septal atrial roof, is muscular and well-trabeculated. It therefore provides a stable position for lead fixation and protection from perforation. In general, it is suggested that BB-P would improve atrial conduction via atrial resynchronization by mimicking the physiologic conduction system via stimulation close to the Sinus Node. BB-P leads to shorter stimulated p-waves and PQ-times. Additionally, stimulation of the atrium follows a craniocaudal direction, which should provide a hemodynamic advantage due to homogeneous atrial depolarization and less interatrial desynchronization. BB-P should therefore have an antiarrhythmic effect leading to the prevention of atrial fibrillation as opposed to the most commonly used Non-BB-P [3,4].

The main risk factors for the incidence of AF in patients requiring atrial pacing are, in addition to classical risk factors such as arterial hypertension or coronary heart disease, the delay of inter-atrial conduction, a prolonged p-wave duration, shortened atrial refractoriness and an increased time for potential AF triggering ectopias [3]. Non-BB-P promotes these factors. Previous studies showed that electrophysiological stimulation at the right atrial appendage leads to a flattening of the p-wave, which also widens and becomes polyphasic [3]. Additionally, Non-BB-P increases PQ-times with delayed atrioventricular conduction and dyssynchronous atrial contraction. Left atrial conduction may be delayed to such an extent that it occurs simultaneously with ventricular pacing. This may result in late diastolic mitral regurgitation, shortening of diastole and diastolic dysfunction. This can lead to a direct proarrhythmic effect. In summary, the risk of atrial fibrillation with atrial pacing via Non-BB-P is most likely proportional to the duration of the stimulated P wave [3,5].

Since the clinical impact of these theoretical benefits of BB-P vs. Non-BB-P in patients with a DDD-pacemaker has not been investigated yet, we sought to address this issue by comparing 302 consecutive patients with BB-P and Non-BB-P. We focused on the endpoints of atrial fibrillation and echocardiographic parameters in our analysis. Additionally, we compared p-wave durations between the two groups.

## 2. Materials and Methods

### 2.1. Study Design

This study is a retrospective observational study that was reviewed and approved by the ethics committee of the Aerztekammer North Rhine-Westphalia (Nr.: 205/2021). The local ethical board of the Aerztekammer North Rhine-Westphalia stated that for this purely retrospective analysis, no board approval was necessary and waived the necessity for informed consent. This study was performed in compliance with the Helsinki Declaration, as revised in 2013. The primary endpoints of this study were the occurrence of atrial fibrillation, the duration of the stimulated p-wave and echocardiographic parameters.

### 2.2. Study Population

A total of 302 consecutive patients who received a DDD-pacemaker for existing sick sinus syndrome or AV block between 2011 and 2013 at the Clinical Department of Cardiology of the Maria Hilf Hospital, Moenchengladbach, Germany, were screened. One patient was excluded due to inconsistent data. Subsequently, 301 consecutive patients with DDD pacemaker implantation were included. The patients were retrospectively divided into two groups based on the location of the atrial leads. Either the atrial electrode was positioned in the right atrial appendage (n = 70), on the anterior wall (n = 61), on the lateral wall (n = 1) (n = 132, Non-BB-P) or near the Bachmann bundle/posterior septal position (n = 169, BB-P).

### 2.3. Pacemaker Implantation

**Figure 1:**
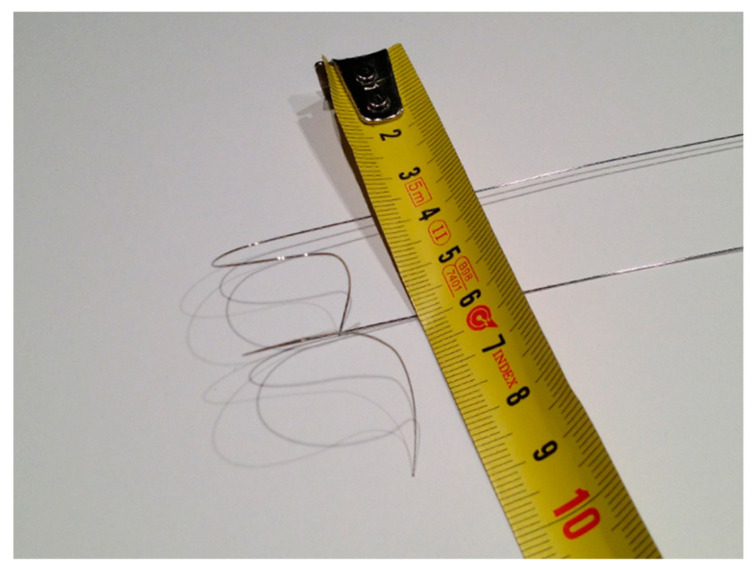
Modification of the standard guide wire of the atrial pacemaker electrode.

**Figure 2:**
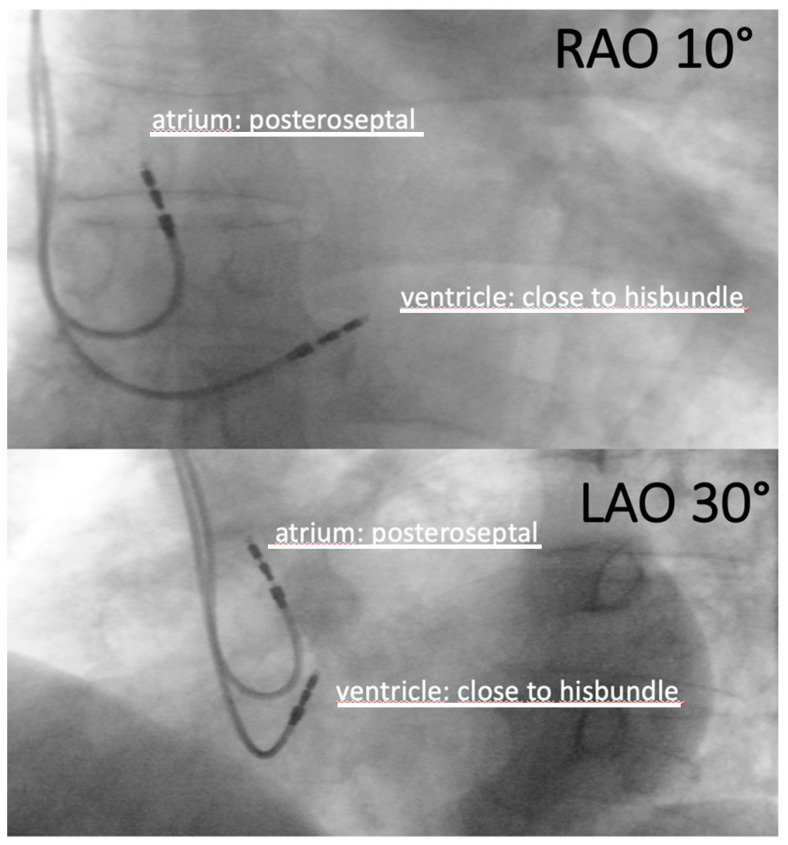
Lead placement in the posteroseptal position of the right atrium. RAO, right anterior oblique; LAO, left anterior oblique.

Placement of the leads was performed as prescribed previously [3,4]. Briefly, we modified the standard guide wire of the atrial pacemaker electrode (Medtronic 5076-52, Dublin, Ireland or Biotronik Solia S 53, Berlin, Germany) according to a specific 3D morphology, as illustrated in Figure 1. Then, we introduced this guide wire into the electrode working channel and maneuvered it into a posteroseptal position using a.p., RAO 10° and LAO 30° fluoroscopy. Lead placement in the posteroseptal position was verified in the LAO 30°, as illustrated in Figure 2, and the electrode was screwed into the atrial myocardium. Complete screwing was verified using fluoroscopy in LAO 30°. The specific preformed guide wire was then removed from the electrode, and the stable lead position was verified using a straight 52 cm guide wire. After successful confirmation of stable posteroseptal electrode implantation, the electrode was fixed on the sleeve using 2.0. Mersilene sutures. Finally, stimulated p-wave duration in the ecg monitoring was compared to intrinsic p-wave duration (maclab software, GE-Healthcare, Boston, MA, USA). The rest of the procedure was performed, as previously described, according to current guidelines and best clinical practice [6]. We used pacemakers from various manufacturers, such as Biotronik (Biotronik, Berlin, Germany) or Medtronic (Medtronic 5076-52, Dublin, Ireland). The decision whether to use Non-BB-P or BB-P in a specific patient was dependent on either the physician’s expertise, since BB-P is more challenging to achieve, or the stability of the electrode position in BB-P patients during implantation. Sometimes, BB-P could not be achieved; therefore, Non-BB-P was used. In general, BB-P was preferably used in patients with an expected high atrial stimulation percentage, such as sick sinus syndrome.

### 2.4. Data Collection

The source of the medical records, samples, data regarding device interrogation, echocardiography, medication and results from diagnostic tests, as well as the history of concomitant diseases, was the patient database of the Kliniken Maria Hilf GmbH, Moenchengladbach (imedONE, Telekom-Healthcare, Frankfurt am Main, Germany). Additionally, we included all available data from general practitioners. The pacemaker follow-up parameters were collected retrospectively after one year at the study center by practicing cardiologists, with a focus on atrial arrhythmias. In detail, echocardiography was performed on multiple machines by certified expert operators. LV function was analyzed using the biplane Simpson method. Cardiac anatomy and function parameters were analyzed as described in current echocardiography guidelines [7]. Specifically, right and left atrial volume were corrected for body surface measured in the 4-chamber view during late systole. Echocardiography was performed 89 days (Min: 1 day, Max: 364 days) after pacemaker implantation. Atrial mode switch was programmed to occur at 175 bpm. Programming of DDD-pacemakers was performed as outlined in current guidelines [6].

### 2.5. Statistical Analysis

The statistical data analysis was performed using Excel and SPSS (version 23.0, IBM, Armonk, NY, USA). Depending on the standard distribution, either the median with its interquartile range, arithmetic mean with its associated standard deviation or percentage were used. The distribution of continuous data was determined by the Kolmogorov–Smirnov test. Further statistical testing was adjusted for the data using the Wilcoxon sign rank test, Exact Fisher test (chi-quadrant test) and Mann–Whitney U test. For age and BMI, the student’s *t*-test was used. Due to incomplete datasets in this real-world analysis, individual n-numbers are provided for each analyzed parameter whenever necessary. A *p*-value of <0.05 was considered statistically significant.

## 3. Results

### 3.1. Baseline Characteristics

A total of 301 patients were included in this study. Pacemakers were implanted from 2011 to 2013 at the Maria Hilf hospital in Moenchengladbach. These patients were divided into two groups as follows: 132 patients were assigned to the group with Non-BB-P and 169 were assigned to the group with BB-P. The mean age in the BB-P group was 76 ± 10 years, 46.7% female, as compared to a mean age of 77.6 ± 9 years, 50% female, in the Non-BB-P group. The baseline characteristics, percentage distribution of comorbidities and echocardiographic data at the time prior to pacemaker implantation of the enrolled patients are shown in Table 1 and Table 2 for each group. For comorbidities and echocardiographic parameters, both groups were homogeneously distributed. There were no statistically significant differences regarding the indications for pacing as well, as shown in Table 3.

### 3.2. Electrophysiological Characteristics

**Figure 3:**
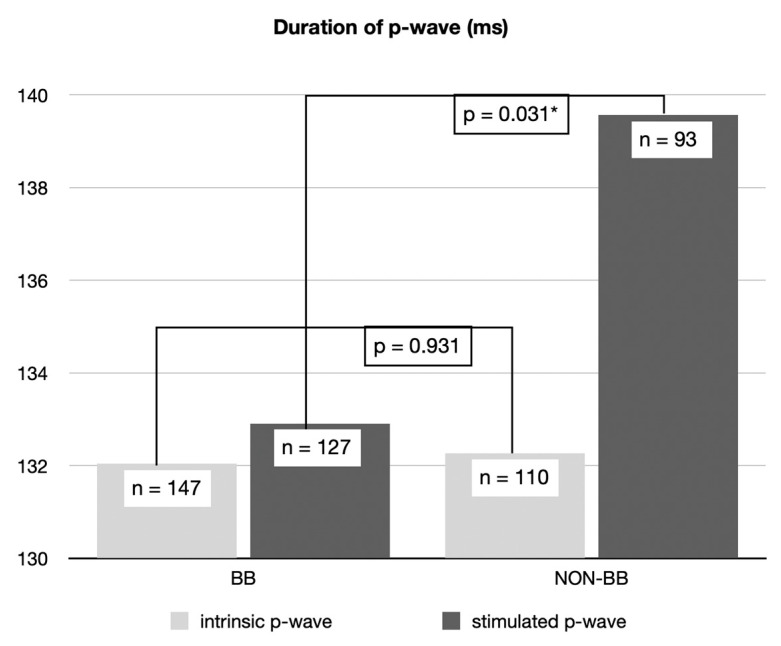
Duration of the p-wave after implantation (ms). Intrinsic: BB-P vs. Non-BB-P, *p* = n.s. Stimulated: BB-P vs. Non-BB-P, *p* = 0.031 (n = number of patients in each group).* = statistically significant.

**Figure 4:**
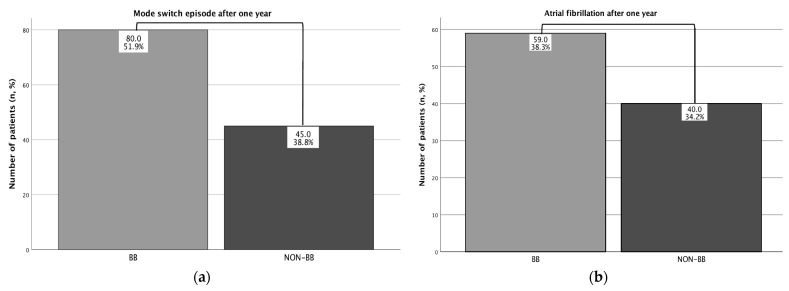
Atrial arrhythmias of BB-P vs. Non-BB-P patients after pacemaker implantation in each group of the crosstable, n = number of patients. (**a**) Mode switch episode after 1 year, *p* = 0.032. (**b**) Atrial fibrillation after one year, *p* = 0.485.

The electrophysical parameters of both groups are shown in Table 4 and Table 5, as well as in Figure 3 and Figure 4. The heart rate as well as the atrial stimulation percentage showed no statistically significant differences in BB-P vs. Non-BB-P. We detected an increased ventricular stimulation percentage in the Non-BB-P group vs. the BB-P group (Non-BB-P: 56.6 ± 42.3%, median: 56.6%, n = 88 vs. BB-P: 45.8 ± 42.7%, median: 33.2%, n = 127, *p* = 0.018).

The intrinsic p-wave duration of the overall cohort was 132.1 ± 22.4 ms, n = 257. In detail, the mean intrinsic p-wave duration in the BB-P group was 132.0 ± 22.0 ms, n = 147, in comparison to 132.3 ± 23.1 ms, n = 110, *p* = n.s., in patients treated with Non-BB-P. This ensured comparability between the groups. The BB-P patients had a significantly shorter stimulated p-wave duration compared to the Non-BB-P patients (BB-P: 132.9 ± 23.7 ms, n = 127 vs. Non-BB-P: 139.6 ± 23.4 ms, n = 93, *p* = 0.031), as shown in Figure 3. Additionally, we found no significant increase in the stimulated p-wave duration in comparison to the intrinsic p-wave in the patients with BB-P (BB-P intrinsic: 131.0 ms +/− 20.8 ms, n = 115 vs. BB-P stimulated: 132.6 ± 22.9 ms, n = 115 *p* = n.s.). Please note that for this analysis, only n = 115 patients in the BB-P group could be used since these BB-P patients had values both for stimulated and intrinsic p-wave duration. This underlines the physiologic stimulation as well as a successful implantation regime in this study. Finally, we were able to show that in the Non-BB-P patients, the stimulated p-waves were significantly longer vs. their intrinsic p-waves (Non-BB-P intrinsic: 131.9 ± 23.8 ms, n = 78 vs. Non-BB-P stimulated: 140.7 ± 24.4, n = 78 *p* = 0.041), indicating potential proarrhythmic effects. Please note that for this analysis, only n = 78 patients in the Non-BB-P group could be used since these Non-BB-P patients had values both for stimulated and intrinsic p-wave duration.

### 3.3. Atrial Arrhythmias Post-Implantation

The evaluation of the pacemaker follow-up examinations in this study neither showed a statistically significant impact of the atrial lead placement on the incidence of atrial fibrillation after one year nor the occurrence of atrial high-frequency episodes. Additionally, interrogation of the pacemaker revealed a significant difference with regard to mode switch episodes in Non-BB-P vs. BB-P (Non-BB-P: 45 (38.8%), n = 116 vs. BB-P: 80 (51.9%), n = 154, *p* = 0.032, Table 5, Figure 4).

### 3.4. Functional Echocardiographic Parameters

The analysis of functional echocardiographic parameters showed no statistically significant differences in BB-P vs. Non-BB-P (Table 6).

### 3.5. Complications–Outcome

We differentiated between early electrode dislocation within 24 h after implantation and late electrode dislocation after 24 h or hospital discharge. Other complications, such as hematoma or pneumothorax, were summarized as other complications. There were no statistically significant differences in complications between the BB-P and Non-BB-P patients (Table 7).

### 3.6. Operative Procedure Data

We found no statistically significant difference in procedure or fluoroscopy dose in the BB-P vs. Non-BB-P patients. Additionally, we found no significant difference in postoperative sensing, impedance and threshold in both groups (Table 8).

## 4. Discussion

The optimal position of the atrial lead in patients with dual-chamber pacemakers is under constant debate. Especially in the field of Bachman bundle stimulation and its impact on relevant clinical endpoints, data are still scarce. Few previous studies suggested a benefit of BB-P [1,8]. With regard to the electrophysiological parameters, our study showed a significant shortening of the stimulated p-wave in patients treated with BB-P vs. Non-BB-P. Additionally, we found that stimulated p-waves in BB-P were not significantly longer as compared to their intrinsic p-waves, while the opposite effect was evident in Non-BB-P. This underlines the almost physiologic stimulation in our BB-P group, while potentially proarrhythmic characteristics were evident in the Non-BB-P group. In summary, these findings are in accordance with previous studies and show that we achieved an adequate stimulation of the Bachman bundle using our implantation technique [8].

However, our electrophysiological findings did not lead to a reduced incidence of atrial fibrillation after a follow-up period of one year in our BB-P group. Existing meta-analyses and retrospective studies suggest that the position of the electrodes for atrial stimulation has an impact after a follow-up period of two years [3,4]. Unfortunately, we were not able to extend our follow-up due to a lack of specific follow-up data. Furthermore, the study by Israel et al. describes that BB-P has a small impact on the incidence of atrial fibrillation when the atrial pacing percentage is less than 50 percent. Since the atrial stimulation percentage in our overall cohort was 36.4%, this could also explain our clinical findings. Finally, our implantation technique did not include the detection of a specific vector analysis of the stimulated p-wave during implantation in combination with fluoroscopy characteristics, as suggested by Infeld et al. Interestingly, these authors were able to detect a decreased incidence of atrial fibrillation with an atrial stimulation percentage of >20% with a 2-year follow-up period [4]. In summary, a positive antiarrhythmic effect of BB-P is suspected, but at the present time, no evidence-based recommendation for a preferred atrial lead position, in addition to the conventional Non-BB-P position, can be made since data from prospective randomized studies are lacking. In our study, we were able to show improved electrophysiological parameters without clinical impact on atrial fibrillation incidence within our follow-up. However, we were also able to detect an increased occurrence of mode switch episodes in BB-P vs. Non-BB-P, hinting at a potential proarrhythmic effect of BB-P within our study.

Finally, we also found a significantly higher ventricular stimulation percentage in the Non-BB-P group vs. BB-P group. One might hypothesize that this is due to the improved atrial conduction in BB-P. An increased ventricular stimulation percentage might trigger more adverse outcomes in the Non-BB-P group over time; however, we were unfortunately not able to extend our follow-up period due to a lack of data. Regarding the complication rate, we were not able to detect a significant difference in BB-P vs. Non-BB-P.

Therefore, selecting patients that would most likely benefit from a more physiological transvenous stimulation, such as BB-P, remains challenging. Recent data on patients implanted with single-chamber leadless pacemakers showed a not negligible incidence of new-onset atrial fibrillation of 6.5% after a 12-month follow-up and 14.1% 24 months after implantation [9,10]. Mitacchione et al. described a reduced incidence of adverse events while conversely achieving a less “physiological” cardiac stimulation with leadless pacers after extraction of transvenous leads. Possibly, patients who are expected to have a very high atrial stimulation rate of over 50%, e.g., in severe sick sinus syndrome, might profit from BB-P.

In summary, we were not able to demonstrate a clear clinical benefit of BB-P vs. Non-BB-P with regard to the incidence of atrial fibrillation or echocardiographic parameters within our follow-up period. The electrophysiological data showed a divergent picture, with either proarrhythmic findings, such as an increased number of mode switch episodes, and potentially beneficial observations, such as a decreased ventricular stimulation percentage in BB-P vs. Non-BB-P patients.

### Limitations

We acknowledge several limitations of this study. This study presents the experience of a single center with a limited sample size (n = 302), which may have not reflected general practice. It was also carried out retrospectively, which, in turn, can have well-known disadvantages, such as survey errors and information bias. Also, existing studies recommend a follow-up period of two years [3,4].

## 5. Conclusions

The findings of our study suggest that in patients requiring dual-chamber implantation, the position of the atrial lead seems not to affect left ventricular function parameters or the risk for the occurrence of atrial fibrillation within a 1-year follow-up. However, pacing in BB-P seems to have a favorable impact on cardiac conduction without a clear clinical impact. Further studies need to investigate the impact of pacing in BB-P on clinical outcomes.

## Figures and Tables

**Table 1:** Baseline characteristics/medical history of both groups at the time prior to pacemaker implantation.

	BB-P (n = 169)	Non-BB-P (n = 132)	*p*-Value
Female sex (n, %)	79 (46.7%)	66 (50.0%)	0.575
Age, years (mean ± SD, median)	76.0 ± 10.1, 77.7	77.6 ± 9.1, 79.5	0.163 ^3^
BMI ^1^, kg/m^2^ (mean ± SD, median)	28.3 ± 8.4, 26.0 (n = 113)	27.9 ± 6.0, 27.0 (n = 86)	0.173 ^3^
Coronary heart disease (n, %)	63 (37.3%)	40 (30.3%)	0.206
Arterial hypertension (n, %)	137 (81.1%)	107 (81.1%)	0.999
Heart failure (n, %)	26 (15.4%)	15 (11.4%)	0.313
Heart surgery (n, %)	30 (17.8%)	15 (11.4%)	0.123
Valve disease (n, %)	7 (4.1%)	1 (0.8%)	0.070
PAD ^2^ (n, %)	8 (4.7%)	5 (3.8%)	0.689
Diabetes mellitus (n, %)	46 (27.2%)	40 (30.3%)	0.557
Chronic obstructive lung disease (n, %)	25 (14.8%)	15 (11.4%)	0.384
Hyperthyroidism (n, %)	6 (3.6%)	9 (6.8%)	0.196
Renal insufficiency (n, %)	29 (17.2%)	22 (16.7%)	0.910

Legend: ^1^ BMI = Body Mass Index; ^2^ PAD = peripheral arterial disease; ^3^ Mann–Whitney U test; all others, chi^2^ test.

**Table 2:** Echocardiographic parameters at baseline before implantation.

	BB-P	Non-BB-P	*p*-Value
LV-EF ^1^ (%, mean ± SD, median)	58.5 ± 6.7; 60.0 (n = 102)	58.6 ± 6.5; 60.0 (n = 82)	0.701 ^2^
Right atrial dilatation (n, %)	15 (15.2%) (n = 99)	11 (13.4%) (n = 82)	0.740
Left atrial dilatation (n, %)	39 (39.4%) (n = 99)	31 (37.8%) (n = 82)	0.827
Diastolic dysfunction (n, %)	32 (33.3%) (n = 96)	24 (30.4%) (n = 70)	0.677

Legend: ^1^ LV-EF = left ventricular ejection fraction; ^2^ Mann–Whitney U test; all others: chi^2^ test; individual n-numbers for the analyzed parameters are presented in each row.

**Table 3:** Indications of pacing.

	BB-P (n = 169)	Non-BB-P (n = 132)	*p*-Value
AV conduction disorders (n, %)	100 (59.2%)	87 (65.9%)	0.446 ^1^
Sinus node disorders (n, %)	67 (39.6%)	43 (32.6%)	
Carotid sinus syndrome (n, %)	2 (1.2%)	2 (1.5%)	

Legend: ^1^ chi^2^ test.

**Table 4:** Pacemaker functional characteristics and electrophysiological data of both groups.

	BB-P	Non-BB-P	*p*-Value
Ventricular stimulation (%, mean ± SD, median)	45.8 ± 42.7, 33.2 (n = 127)	56.6 ± 42.3, 56.6 (n = 88)	0.018 *
Atrial stimulation (%, mean ± SD, median)	37.0 ± 33.3, 28.9 (n = 121)	35.5 ± 33.1, 20.0 (n = 82)	0.755
Duration intrinsic p-wave (ms; mean ± SD, median)	132.0 ± 22.0, 130.0 (n = 147)	132.3 ± 23.1, 130.0 (n = 110)	0.931
Duration stimulated p-wave (ms, mean ± SD; median)	132.9 ± 23.7, 130.0 (n = 127)	139.6 ± 23.4, 140.0 (n = 93)	0.031 *
Duration intrinsic QRS complex (ms, mean ± SD, median)	117.5 ± 30.6, 110.0 (n = 164)	110.7 ± 26.7, 100.0 (n = 121)	0.075
Duration stimulated QRS complex (ms, mean ± SD, median)	158.2 ± 26.3, 160.0 (n = 153)	153.3 ± 21.1, 160.0 (n = 112)	0.074

Legend: * sig difference in Mann–Whitney U test; individual n-numbers for the analyzed parameters are presented in each row.

**Table 5:** Heart rate and atrial arrhythmias of both groups after pacemaker implantation.

	BB-P	Non-BB-P	*p*-Value
Heart rate (bpm, mean ± SD, median)	58.5 ± 4.0, 60.0 (n = 169)	58.9 ± 3.6, 60.0 (n = 131)	0.288
Atrial high-frequency episodes (n, %)	51 (34.7%) (n = 147)	35 (31.0%) (n = 113)	0.527
Mode switch episode (n, %)	80 (51.9%) (n = 154)	45 (38.8.%) (n = 116)	0.032 *
Atrial fibrillation after one year (n, %)	59 (38.3%) (n = 154)	40 (34.2%) (n = 117)	0.485

Legend: * sig., chi^2^ test; individual n-numbers for the analyzed parameters are presented in each row.

**Table 6:** Echocardiographic parameters one year after pacemaker implantation.

	BB-P	Non-BB-P	*p*-Value
LV-EF after 6 months (%, mean ± SD, median)	57.1 ± 8.4, 60.0 (n = 60)	56.0 ± 9.6, 60.0 (n = 45)	0.525 ^1^
Right atrial dilatation (n, %)	19 (27.9%) (n = 68)	14 (28.0%) (n = 50)	0.994
Left atrial dilatation (n, %)	40 (58.8%) (n = 68)	21 (42.0%) (n = 50)	0.071
Diastolic dysfunction (n, %)	37 (55.2%) (n = 67)	18 (38.3%) (n = 47)	0.075

Legend: ^1^ Mann–Whitney U test; all others: chi^2^ test; individual n-numbers for the analyzed parameters are presented in each row.

**Table 7:** Complications after pacemaker implantation between the two groups.

Complications	BB-P (n = 169)	Non-BB-P (n = 132)	*p*-Value
Early electrode dislocation (n, %)	1 (0.6%)	1 (0.8%)	0.860
Late electrode dislocation (n, %)	2 (1.2%)	0	0.210
Other complications (n, %)	0	1 (0.8%)	0.257
No hospitalization after one year (n, %)	142 (84.0%)	104 (78.8%)	0.243

Legend: chi^2^ test.

**Table 8:** Operative procedure data.

	BB-P	Non-BB-P	*p*-Value
Procedure Time (min., mean ± SD, median)	70.2 ± 21.4, 65.0 (n = 169)	74.1 ± 23.0, 70.0 (n = 132)	0.135
Fluroscopy Dose (cGycm^2^, mean ± SD, median)	452.0 ± 443.0, 323.1 (n = 167)	424.2 ± 292.7, 383.9 (n = 132)	0.232
Threshold, Impulse Amplitude (V, mean ± SD, median)	0.68 ± 0.54, 0.5 (n = 154)	0.63 ± 0.33, 0.5 (n = 123)	0.911
Threshold, Impulse Width, (V, mean ± SD, median)	0.46 ± 0.05, 0.5 (n = 154)	0.45 ± 0.05, 0.5 (n = 123)	0.382
Sensing (mV, mean ± SD, median)	3.8 ± 2.0, 3.5 (n = 21)	3.0 ± 2.3, 2.3 (n = 6)	0.476
Impedance (Ohm, mean ± SD, median)	443.8 ± 111.8, 427.0 (n = 60)	440.8 ± 77.1, 440.0 (n = 48)	0.643

Legend: Mann–Whitney U test; individual n-numbers for the analyzed parameters are presented in each row.

## Data Availability

The original contributions presented in this study are included in this article. Further inquiries can be directed to the corresponding authors.
